# Chromosomal analysis of progenies between *Lilium* intersectional hybrids and wild species using ND-FISH and GISH

**DOI:** 10.3389/fpls.2024.1461798

**Published:** 2024-10-22

**Authors:** Mei Zhou, Xue Yong, Jungang Zhu, Qian Xu, Xiaodan Liu, Lu Zhang, Lisha Mou, Lijia Zeng, Mengxi Wu, Beibei Jiang, Yin Jia, Peihua Zhang, Yuanzhi Pan

**Affiliations:** ^1^ College of Landscape Architecture, Sichuan Agricultural University, Chengdu, China; ^2^ Chengdu Botanical Garden, Chengdu, Sichuan, China; ^3^ Floriculture Research Institute, Yunnan Academy of Agricultural Sciences National Engineering Research Center for Ornamental Horticulture, Key Laboratory for Flower Breeding of Yunnan Province, Kunming, China; ^4^ College of Forestry, Sichuan Agricultural University, Chengdu, China

**Keywords:** *Lilium*, intersectional hybrids, wild species, cytogenetics, ND-FISH, GISH, oligonucleotide probes

## Abstract

**Introduction:**

Intersectional hybrids in lilies possess significant breeding value, but the lack of complete lily genomes and complex genotypes pose challenges for early identification of lily hybrids. This study aimed to use intersectional hybrid cultivars as female parents and wild lilies as male parents to facilitate early identification of hybrid offsprings and enhance the efficiency and convenience of the process.

**Methods:**

We investigated the nature of cross combinations using Non-denaturing Fluorescence In Situ Hybridization (ND-FISH) and Genomic In Situ Hybridization (GISH) techniques. Three novel oligonucleotide probes—Oligo-pTa794, Oligo-pITS and Oligo-telo—were developed for lily chromosome research.

**Results:**

Our results demonstrated successful hybridization between wild lilies and intersectional hybrid cultivars, producing a total of 130 hybrid progenies. The combination of ND-FISH and GISH analyses effectively revealed the genomic composition of the hybrid progeny and determined the parental origin of specific chromosomes.

**Discussion:**

This research provides significant guidance for lily breeding practices and offers a valuable reference for the application of ND-FISH and GISH techniques in interspecific hybridization breeding and molecular cytogenetic research across various plant species. The methods developed enable more precise, efficient, and convenient identification of hybrid offsprings.

## 1 Introduction

Lily, comprising approximately 100 wild species within the classification of seven sections, exhibits extensive phenotypic and genetic diversity as a horticulturally important flower within the genus *Lilium* (De Jong, 1974; Duan et al., 2022). Due to these unique characteristics in wide geographical distribution, species richness, and intricate hybridization history, breeding programs and genomic analyses of genus *Lilium* are quite challenging (Lim et al., 2008; Du et al., 2014; Kim et al., 2022). Most of today’s lily cultivars are generated by intra-sectional and inter-sectional hybrids (Van Tuyl and Arens, 2011). Intersectional hybrids often exhibit closer genetic relationships with various species and cultivars compared to some intrasectional hybrids, potentially reducing the difficulties associated with hybridization breeding. This genetic proximity can facilitate more successful hybridization and increase the likelihood of obtaining viable progenies with desired trait combinations (Marasek-Ciolakowska et al., 2018). These hybrids can successfully serve as maternal parents, which is attributed to the presence of Fritillaria-type embryo sacs (Zhou et al., 2014; Cao et al., 2019; Cui et al., 2022) and the 2n gametes (Chung et al., 2013; Liu et al., 2021; Xie et al., 2022). Hence, utilizing interspecific hybridization as parental lines possess high breeding value for cultivar improvement in enhanced resistance, improved ornamental characteristics, and adaptability (Lim et al., 2008; Khan et al., 2012).

Hybrid identification in lilies is still quite difficult due to the complex genotypes resulting from intersectional hybridization and the long juvenile phase of lilies hindering traditional morphological identification methods (Jie et al., 2009). Sequence-independent molecular DNA markers such as AFLP and SSR have been successfully addressed such issue and are widely applied in the early identification of lily hybrids (Yin et al., 2013; Du et al., 2015). Nevertheless, with the lack of a complete reference genome for lilies, AFLP and SSR struggle to accurately distinguish contributions from different genomes in complex polyploids and wide intersectional hybrids, leading to difficulties in the precision of result interpretation. Cytogenetic techniques, particularly Fluorescence *In Situ* Hybridization (FISH) and Genomic *In Situ* Hybridization (GISH), have emerged as powerful tools for lily hybrid identification (Marasek et al., 2004; Xie et al., 2010; Liu et al., 2023; Xiao et al., 2023). These techniques offer direct visualization of genomic composition at the chromosomal level, providing more intuitive and detailed information for complex lily hybrid analysis (Xiao, 2019; Islam et al., 2022). However, traditional FISH techniques are restrained by their complex procedures, high costs, and potential for chromosome morphology alterations under high temperatures (Sun et al., 2022).

In comparison with FISH, Non-Denaturing Fluorescence (ND-FISH) simplifies the procedure and shortens hybridization time (Fu et al., 2015). It utilizes oligonucleotide probes, which can be synthetically produced, precisely designed for enhanced specificity, and stored long-term due to their stability. ND-FISH has been widely applied in other plants with outstanding performances (Yu et al., 2021; Zou et al., 2021; Jiang et al., 2023). Thus, the combination of ND-FISH and GISH acts as an efficient solution to the limitation of traditional FISH, enabling simultaneous analyses in the same division phase, further enhancing efficiency and accuracy (Cuadrado and Jouve, 2010). Despite the fact that the combination of ND-FISH and GISH is a promising technique in hybrid identification in lilies, it remains largely unexplored and requires in-depth investigations to fully harness its high potentials.

To this end, this study investigated the applicability of oligonucleotide probes to lily cytogenetic studies. We combined the use of ND-FISH and GISH techniques, and applied three developed oligonucleotide probes (i.e., Oligo-pTa794, Oligo-pITS, and Oligo-telo) to examine the progeny of crosses between intersectional hybrids (LA and OT: Longiflorum × Asiatic and Oriental × Trumpet) and wild lily species, focusing on early identification of hybrids with complex genetic backgrounds. This strategy provides a powerful tool for detailed genetic analysis in lily breeding programs, potentially accelerating the development of new cultivars with desired traits.

## 2 Materials and methods

### 2.1 Plant materials

Two wild lily species and six intersectional hybrid cultivars were selected for hybridization experiments. The wild species, *Lilium davidii* var. *unicolor* and *Lilium regale* E. H. Wilson, both diploid (2n = 2x = 24), were collected from Wenchuan County, Sichuan, China, and used as male parents. The intersectional hybrid cultivars, supplied by Heidi’s Garden, Sichuan, China, served as female parents and included three LA hybrids (‘Richmond’, ‘Eremo’, and ‘Armandale’) and three OT hybrids (‘Nymph’, ‘Robina’, and ‘Gaucho’). All plant materials were cultivated in the nursery Garden of Sichuan Agricultural University. The plants were irrigated weekly and fertilized biweekly to ensure optimal growth and development.

### 2.2 Pollination and embryo rescue

The pollination and embryo rescue were referred to Zhou et al. (2011), (Zhou et al, 2012), with minor modifications. All cross combinations were conventionally pollinated. The stamens and petals were removed from the female flowers 1d before blooming and wrapped in a sulfuric acid paper bag to prevent self-pollination or contamination from other pollen sources. To avoid asynchronous flowering periods, pollen was collected and stored in advance. At the time of pollination, the pollen was thawed and then applied. Pollination was conducted between 9:00-12:00 AM on the day of flowering, after which the flowers were re-bagged to prevent contamination. Embryo rescue was performed when the fruit started to turn yellow and soften, and the number of seeds with embryos was counted. Young embryos and ovules were isolated and cultured on lily embryo rescue medium (pH=5.8) containing 2.24 g/L MS, 60 g/L sucrose, and 5 g/L gelrite. These were kept in the dark to germinate, then transferred to lily propagation medium (pH=5.8) with 2.24 g/L MS, 50 g/L sucrose, and 5 g/L gelrite at 25°C under 2000 lx light intensity for about 10 weeks.

### 2.3 Chromosome preparation

Chromosome spreads were prepared by the method described in the previous study (Han et al., 2006), with a minor modification.

Young root tips (approximately 2 cm long) were collected from bulbs and treated in a nitrous oxide gas chamber for 2.5 h. The tips were then fixed in 90% acetic acid for 10 min, washed 3-5 times with distilled water, and stored in 70% (v/v) ethanol at -20°C. Prior to use, the root tips were removed from storage and rinsed 3-5 times with distilled water. Approximately 2 mm of top meristem was excised and enzymatically hydrolyzed in 20 μL of enzyme solution (2% cellulase and 1% pectinase mixture) for 60 min at 37°C. Following digestion, the meristems were rinsed and homogenized in 70% ethanol. The resulting tissue fragments were dispersed in 50 μL of glacial acetic acid and placed on glass slides, air-dried overnight at 37°C and then stored at -20°C prior to ND-FISH and GISH analysis. For each hybrid combination, at least three offsprings were analyzed. From each offspring, at least ten well-spread metaphase chromosome slides were prepared and selected for analysis.

### 2.4 Non-denaturing fluorescence *in situ* hybridization

In the ND-FISH experiment, Oligo-pTa794 and Oligo-pITS were selected as oligonucleotide probes used in the ND-FISH experiment based on their proven effectiveness in targeting the non-transcribed spacer (NTS) and internal transcribed spacer (ITS) sequences of wild Sinomartagon lilies during evaluation (Lee et al., 2014). The probe Oligo-pTa794 (5’FAM-5′TCAGAACTCCGCAGTTAAGCGTGCTTGGGCGAGAGTAGTAC3′), containing a 41 bp fragment of 5S rDNA repeat sequence, originally designed for wheat studies (Sanz et al., 2010), the probe Oligo-pITS (5’TAMRA-5′CGCATCGATGAAGAACGTAGCGAAATGCGATACTTGGTGTGAA3′), was derived from 18S-5.8S-28S rDNA sequence, the probe Oligo-Telo (5’Cy5-5′TTTAGGGTTTAGGGTTTAGGG3′) was a telomere-specific probe (Ren et al., 2022), The synthesized oligo probes were diluted with 1×TE buffer (pH=8.0). The signals of probes labeled with 6-FAM, Tamra and Cy5 were displayed in green, red and far-red (shown as yellow in the figures for visual clarity), respectively. The chromosome spreads of materials were prepared through the methods described by Han et al (Han et al., 2006). The probe amount per slide was 1 μL for Oligo-pTa794 and Oligo-pITS each, and 0.5 μL for Telo. The probe mixture (each probe in 2×SSC and 1×TE buffer, pH=7.0, total volume = 10 μL) was dropped at the center of the cell spreads and covered with a glass coverslip. Slides were stored in a moist box at 42°C for 2 h and washed in 2×SSC at room temperature. Chromosomes were counterstained with 4–6-diamino-2-phenylindole (DAPI) solution (Vector Laboratories, Inc., Burlingame, CA, USA) (Fu et al., 2015). Chromosome images were captured with an Olympus BX-51 microscope equipped with a DP-70 CCD camera (Li et al., 2018) and a 60x objective with a numerical aperture (NA) of 1.42. At least five best slides with well-spread metaphase chromosomes were selected for analysis and measured using Adobe Photoshop CS 5.0. The chromosomes were arranged based on decreasing short arm lengths according to Lim et al. (2001).

### 2.5 Genomic *in situ* hybridization

The samples used for ND-FISH analysis were subsequently used for GISH, which were washed with 100% ethanol for 1 min and then placed under bright light for 24 hours. The total genomic DNA of *Lilium longiflorum* Thunb. (2n=2x=24, LL group), *Lilium speciosum* Thunb. var. *‘gloriosoides’* Baker (2n=2x=24, OO group), Asiatic ‘Tresor’ (2n=4x=48, AA group) and *L. regale* (2n=2x=24, TT group) was extracted from fresh leaves using CTAB method (Murray and Thompson, 1980).

*Lilium longiflorum* (LL) and *L. speciousm ‘gloriosoides’* genomic DNA labeled with dUTP-ATTO-550 (Jena Bioscience, Jena, Germany) via nick translation was used as the probe, Asiatic ‘Tresor’ and *L. regale* (TT) DNA was used as the blocker (ratio of 1:150). The GISH analysis was performed according to a published method that was modified slightly (Gong et al., 2023). Briefly, 1 µL probe and 9 µL hybridization mixture (1 g dextran sulfate, 5 mL formamide, 1 mL 20×SSC, 1 mL salmon sperm, and 2 mL ddH_2_O) were mixed and heated at 95°C for 10 minutes. Then, 3 μL of blocker was added and mixed thoroughly before dispensing 13 μL of the mixture onto the slides. The samples were denatured at 85°C for 5 min and then incubated overnight at 50°C. They were subsequently washed with 2× SSC at 50°C for 20 min and then with ddH_2_O for 1 min and 100% ethanol for 1 min. Chromosomes were counterstained with 4–6-diamino-2-phenylindole (DAPI) solution.

The software and techniques used for observing and processing chromosome images in GISH technology are consistent with those utilized in the aforementioned ND-FISH technology. The original cytogenetic images displayed in this study were captured under the same conditions and parameters of the fluorescence microscope. ImageJ software was used to measure the length of chromosomes.

## 3 Results

### 3.1 Production of hybrid offsprings

**Figure 1:**
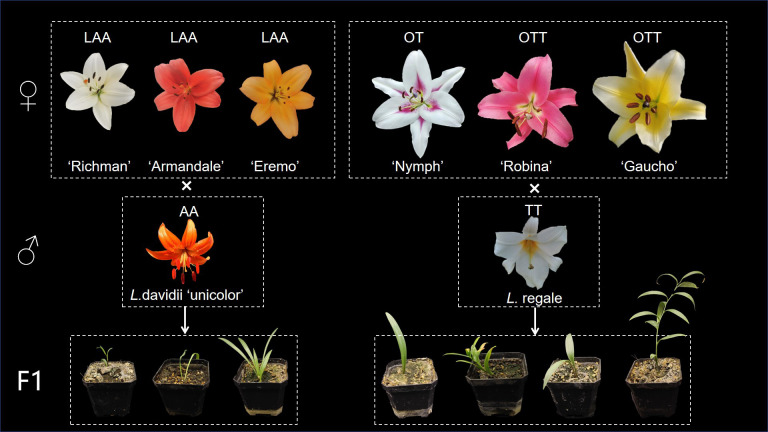
Interspecific hybridization scheme of lily cultivars and wild species.

The distant hybridization methods between intersectional hybrids and two wild Lilium species are illustrated in **Figure 1**, while hybridization results presented in **Table 1**. Progenies were successfully obtained from all hybridization combinations in this study.

**Table 1:** Six hybrid combinations of lilies and their fruiting results.

Female		Male	Flower No.	Swelling ovary No.	Seed with embryo No.	Embryo rate (%)	Seedlings No.
Variety name	Genome type						
‘Richmond’	LAA	*L.davidii* ‘unicolor’	15	35	15	0.43	6
‘Armandale’			10	15	2	0.13	2
‘Eremo’			15	6	2	0.33	2
‘Nymph’	OT	L. regale	50	288	192	0.66	118
‘Robina’	OOT		50	3	1	0.3	1
‘Gaucho’			50	7	3	0.42	1

**Figure 2:**
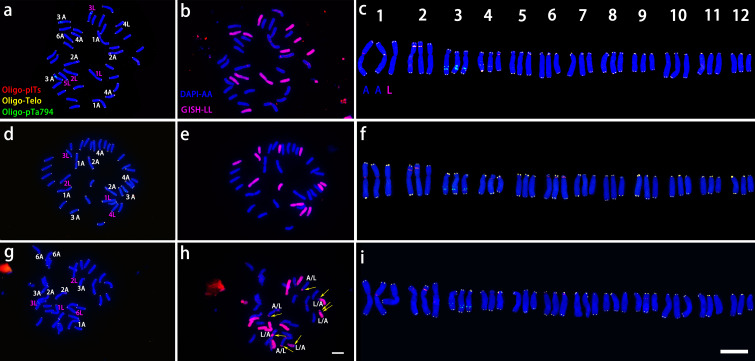
ND-FISH experiments on chromosomes of the three triploid LA lilies were performed using Oligo-pITS (red), Oligo-Telo (yellow) and Oligo-pTa794 (green) as probes, showing the fluorescent signals on the chromosomes of ‘Richmond’ **(A)**, ‘Armandale’ **(D)** and ‘Eremo’ **(G)**. GISH experiments on chromosomes of the three triploid LA lilies were performed with genomic DNA of *L. longiflorum* as a probe showing the chromosome composition of ‘Richmond’ **(B)**, ‘Armandale’ **(E)** and ‘Eremo’ **(H)**. Identified metaphase chromosomes from ‘Richmond’ **(C)**, ‘Armandale’ **(F)** and ‘Eremo’ **(I)** are displayed. Homologous chromosomes were classified according to the signal positions of Oligo-pITS (red), Oligo-Telo (yellow) and Oligo-pTa794 (green) as well as the locations of centromeres and chromosome morphology. Chromosomes are arranged in order of descending short arm length. Chromosomes were counterstained with DAPI (blue). Scale bar 10 μm.

The LA hybrids used as maternal parents were triploid, as shown in **Figure 2**. These triploid LA hybrids, when crossed with the diploid *L. davidii* var. *unicolor*, produced a total of 10 progenies. Among these combinations, the cross using ‘Richmond’ as the maternal parent and *L. davidii* var. *unicolor* as the paternal parent demonstrated the highest embryo sac formation rate and yielded the greatest number of hybrid seedlings. In the interspecific hybridizations of OT lilies, ‘Nymph’ was diploid, while ‘Robina’ and ‘Gaucho’ were triploid (**Figure 2**). Crosses with *L. regale* resulted in a total of 120 hybrid seedlings. The hybridization with ‘Nymph’ as the maternal parent showed the best result, with an embryo sac formation rate of 0.66 and 118 hybrid seedlings were obtained. This success is closely related to the unreduced 2n gametes produced by ‘Nymph’. The ability of these triploid intergroup hybrids to produce progeny is associated with the Fritillaria-type embryo sac of lilies, which can generate euploid endosperms to nourish the survival of aneuploid embryos. When the triploid OT hybrid cultivars ‘Robina’ and ‘Gaucho’ were used as maternal parents in crosses with *L. regale*, the embryo sac formation rate was low (0.3-0.42), resulting in only very few hybrid seedlings.

### 3.2 ND-FISH and GISH analysis of parental materials

All three LA lily hybrids (‘Richmond’, ‘Armandale’, and ‘Eremo’) were triploids (2n=3x=36) with a genomic composition of 24A+12L (**Figure 2**). Chromosomes were arranged in pairs based on the ND-FISH and GISH images, with the two A-genome copies on the left and the L-genome copy on the right (**Figures 2C, F, I**). These triploid LA hybrids shared common signal loci: Oligo-pITS signals were located on the centromeres of the two A-genome copies of chromosome 1, in the subtelomeric regions of the short arms of two A-genome copies of chromosome 2, and on the long arm of the L-genome copy of chromosome 3. Two Oligo-pITS signals were also observed on the long arm of chromosome 3. The L-genome copy of chromosome 3 possessed both Oligo-pITS and Oligo-pTa794 signals. ‘Richmond’ displayed additional distinct Oligo-pITS signals on all three copies of chromosome 4, the L-genome copy of chromosome 5, and one A-genome copy of chromosome 6 (**Figures 2A–C**). Both ‘Armandale’ and ‘Eremo’ exhibited Oligo-pITS signals near the centromere of one A-genome copy, on the short arm of one L-genome copy of chromosome 4, and on the short arms of three copies of chromosome 6 (**Figures 2D–I**). ‘Eremo’ significantly differed by possessing several recombinant chromosomes, classified as A/L and L/A types based on their centromeric regions (**Figure 2B, E, H**). L/A chromosomes contained L. longiflorum centromeres, indicating the introgression of Asiatic genome segments into L. longiflorum chromosomes. Conversely, A/L chromosomes, with Asiatic centromeres, showed intergenomic translocations with L. longiflorum. ‘Eremo’ contained four L/A recombinant chromosomes and three A/L recombinant chromosomes, while no recombination was observed in ‘Richmond’ or ‘Armandale’.

**Figure 3:**
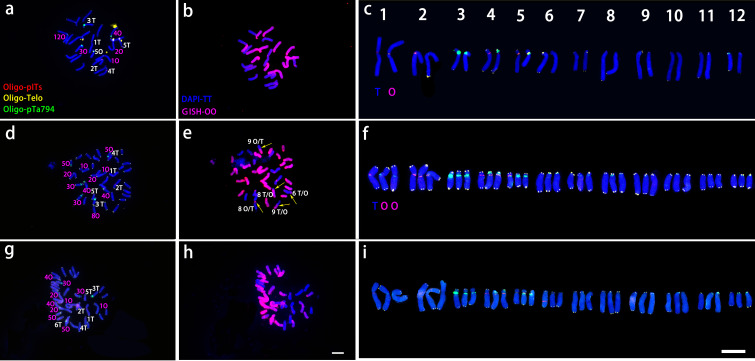
ND-FISH experiments on chromosomes of the three OT lilies were performed using Oligo-pITS (red), Oligo-Telo (yellow) and Oligo-pTa794 (green) as probes, showing the fluorescent signals on the chromosomes of ‘Nymph’ **(A)**, ‘Gaucho’ **(D)** and ‘Robina’ **(G)**. GISH experiments on chromosomes of the three OT lilies were performed with genomic DNA of *L. speciousm ‘gloriosoides’* as a probe showing the chromosome composition of ‘Nymph’ **(B)**, ‘Gaucho’ **(E)**and ‘Robina’ **(D)**. Identified metaphase chromosomes from ‘Nymph’ **(C)**, ‘Gaucho’ **(F)** and ‘Robina’ **(I)** are displayed. Homologous chromosomes were classified according to the signal positions of Oligo-pITS (red), Oligo-Telo (yellow) and Oligo-pTa794 (green) as well as the locations of centromeres and chromosome morphology. Chromosomes are arranged in order of descending short arm length. Chromosomes were counterstained with DAPI (blue). Scale bar 10 μm.

‘Nymph’ was diploid (2n=2x=24) with a genomic composition of 12O+12T, whereas both ‘Robina’ and ‘Gaucho’ were triploid (2n = 3x = 36) with a composition of 24O + 12T (**Figure 3**). Chromosomes were paired according to ND-FISH and GISH images, revealing both similarities and variations among genotypes. In each pair, the T-genome chromosome was positioned on the left, with one or two O-genome copies on the right (**Figures 3C, F, I**). In ‘Nymph’, at least seven Oligo-pITS signals were detected, located near the centromeres of both copies of chromosome 1and 2, in the subtelomeric region of chromosome 4, and near the centromeres of both copies of chromosome 5. Oligo-pTa794 signals were present near the centromeres of both copies of chromosome 3, with the signal on the T-genome copy being more intense than on the O-genome copy), and near the centromeres of T-genome copies of chromosome 5 and 12. The O-genome copy of chromosome 5 contained both Oligo-pITS and Oligo-pTa794 signals (**Figures 3A–C**). ‘Gaucho’ and ‘Robina’ exhibited highly similar oligonucleotide probe signal distributions across various chromosomes. The centromere of T-genome copy of chromosome 1 had one Oligo-pITS signal, while the centromeres of two O-genemo copies of chromosome 1 each had two Oligo-pTa794 signals. The centromeres of all three copies of chromosome 2 carried one Oligo-pITS signal each, while all three copies of chromosome 3 had Oligo-pTa794 signals, with the T-genome copy displaying significantly higher signal intensity than the O-genome copies. The centromere of the T-genome copy of chromosome 4 contained two Oligo-pITS signals, while the O-genome copy of chromosome 4 displayed both Oligo-pITS and Oligo-pTa794 signals. The centromere of T-genome copy of chromosome 5 had one Oligo-pITS signal, whereas the centromeres of two O-genome copies had two Oligo-pTa794 signals (**Figures 3D–I**). Additionally, the long arm of the T-genome copy of chromosome 6 in ‘Robina’ carried one Oligo-pITS signal, possibly marking a secondary constriction. The main difference between ‘Robina’ and ‘Gaucho’ was the occurrence of chromosomal recombination. While ‘Robina’ showed no evidence of recombination, ‘Gaucho’ exhibited two O/T recombinant chromosomes and three T/O recombinant chromosomes (**Figures 3B, E, H**).

**Figure 4:**
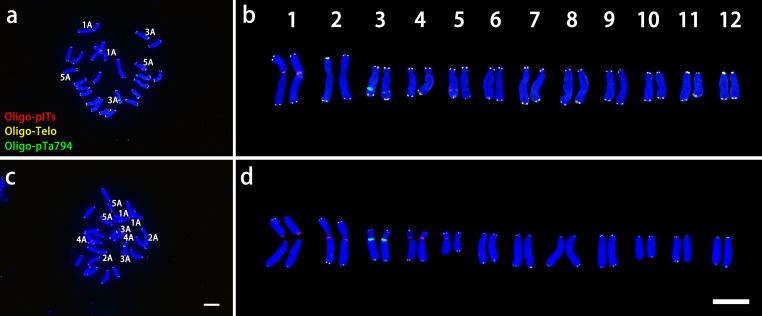
ND-FISH experiments on chromosomes of the two wild species were performed using Oligo-pITS (red), Oligo-Telo (yellow) and Oligo-pTa794 (green) as probes, showing the fluorescent signals on the chromosomes of *L. davidii* var. *unicolor* **(A)** and *L. regale* **(C)**. Identified metaphase chromosomes from *L. davidii* var. *unicolor* **(B)** and *L. regale* **(D)** are displayed. Homologous chromosomes were classified according to the signal positions of Oligo-pITS (red), Oligo-Telo (yellow) and Oligo-pTa794 (green) as well as on centromere locations and chromosome morphology. Chromosomes are arranged in order of descending short arm length. Chromosomes were counterstained with DAPI (blue). Scale bar 10 μm.

As shown in **Figure 4**, In *L. davidii* var. *unicolor* had four Oligo-pITS signals: two on the centromeres of chromosomes 1 and two on the long arms of chromosome 5, Additionally, two Oligo-pTa794 signals were situated on the long arm of chromosome 3. In *L. regale*, eight Oligo-pITS signal were observed near the centromeres of chromosomes 1, 2, 4 and 5 and two Oligo-pTa794 signals were located near the centromeres of chromosome 3 (**Figures 4B, D**).

### 3.3 ND-FISH and GISH analysis of ‘Richmond’× *L. davidii* var. *unicolor*

**Figure 5:**
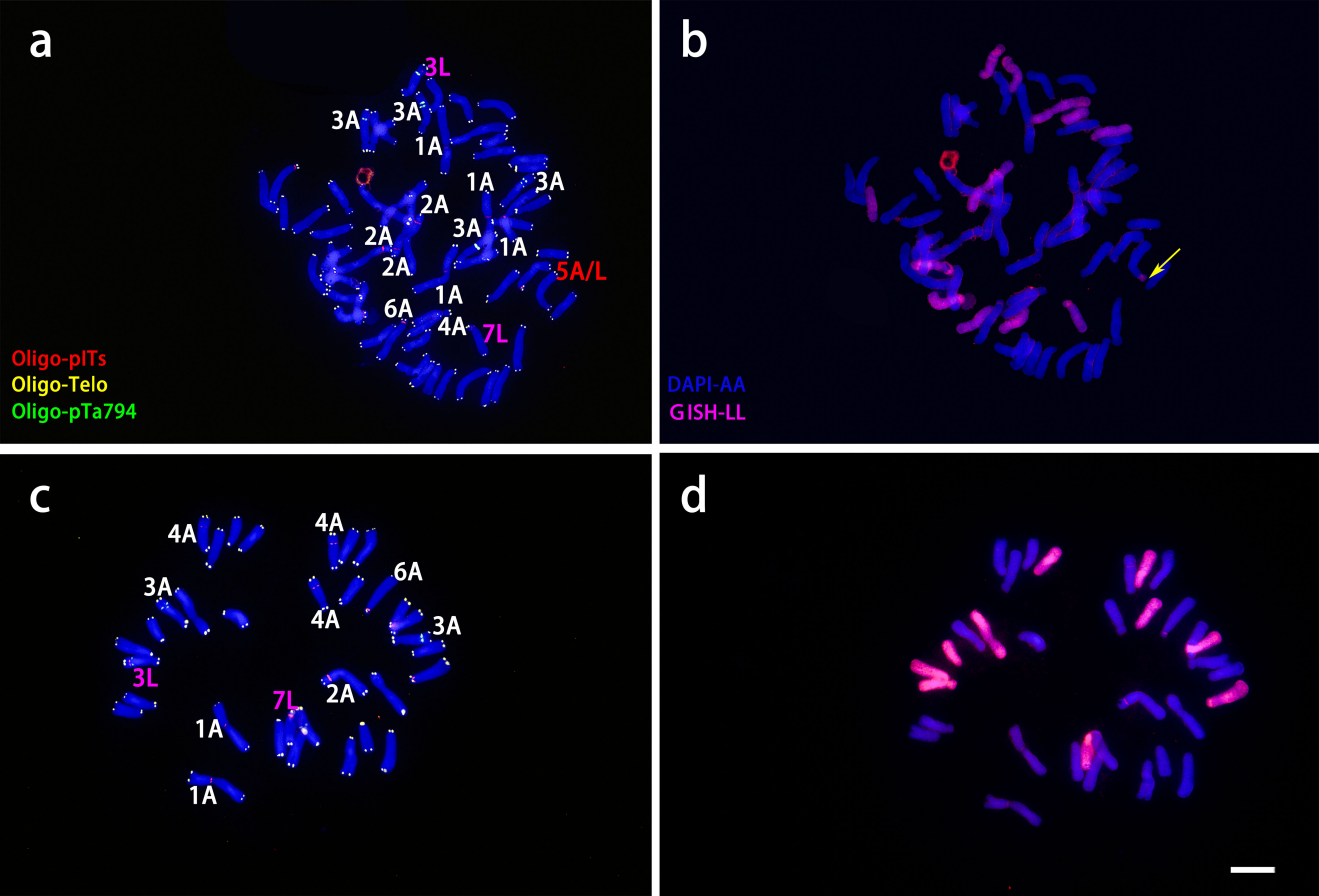
ND-FISH experiments on chromosomes of the two ‘Richmond’ × *L. davidii* var. *unicolor* hybrid progenies were performed using Oligo-pITS (red), Oligo-Telo (yellow) and Oligo-pTa794 (green) as probes, showing the fluorescent signals on the chromosomes of RD-003 **(A)** and RD-005 **(C)**. GISH experiments on chromosomes of the two ‘Richmond’× *L. davidii* var. *unicolor* hybrid progenies were performed with genomic DNA of *L. longiflorum* as a probe showing the chromosome composition of RD-003 **(B)** and RD-005 **(D)**. The yellow arrow points to the break point of the recombinant chromosomes. Chromosomes were counterstained with DAPI (blue). Scale bar 10 μm.

**Figure 6:**
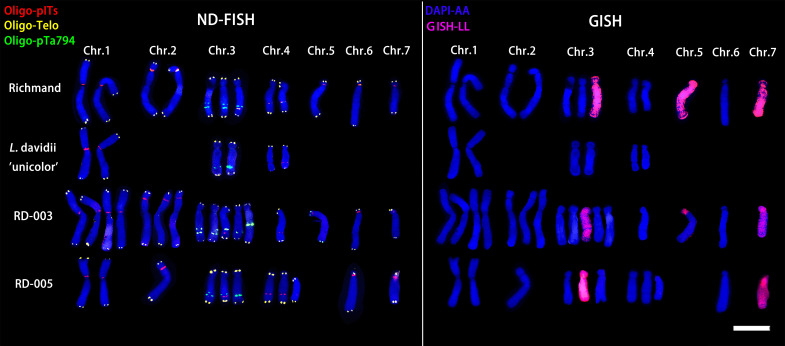
ND-FISH (left) and GISH (right) patterns of partial chromosomes from maternal and paternal parents and two progenies.

ND-FISH and GISH techniques were utilized to analyze the signal loci of Oligo-pITS and Oligo-pTa794 in the hybrid progeny of ‘Richmond’ × *L. davidii* var. *unicolor* (**Figures 5**, **6**). RD-003 and RD-005 hybrids were confirmed to be true hybrids, with chromosomes from both parents being distinctly reflected in the progeny. RD-003 was aneuploid with 58 chromosomes. Chromosome 1 of RD-003 comprised two A-genome copies from ‘Richmond’ and two A-genome copies from *L. davidii* var *unicolor*. Chromosome 2 consisted of two A-genome copies from ‘Richmond’, while the third copy, likely from *L. davidii* var. *unicolor*, lacked Oligo-pITS fluorescence labeling. This absence could be attributed to the base mutations or chromosomal recombination during the hybridization process. Oligo-pTa794 and Oligo-pITS successfully labeled all copies of chromosome 3, which included three copies from the ‘Richmond’ and two from the *L. davidii* var *unicolor*. However, one copy from the *L. davidii* var. *unicolor* underwent genetic recombination, resulting in altered morphology and differences in fluorescence labeling in the progeny compared to the parental chromosome. The origin of chromosome 4 could not be conclusively determined. Chromosome 5 possibly originated from an A-genome copy of the ‘Richmond’ and had undergone recombination. Chromosome 6 was presumed to be inherited from the A-genome of the maternal parent, while chromosome 7 possibly originated from the L-genome copy of the maternal parent. RD-005 was aneuploid with 34 chromosomes. Two A-genome copies of chromosome 1 possibly originated from one A-genome copy of ‘Richmond’ and one A-genome copy of *L. davidii* var. *unicolor*. Chromosome 2 was inherited from an A-genome copy of ‘Richmond’. Chromosome 3 and 4 each contained two copies from ‘Richmond’ and one A-genome copy from *L. davidii* var. *unicolor*. Chromosome 6 was likely derived from the A-genome copy of the ‘Richmond’, while chromosome 7 possibly originated from the L-genome copy of the ‘Richmond’.

### 3.4 ND-FISH and GISH analysis of ‘Nymph’ × *L. regale*

**Figure 7:**
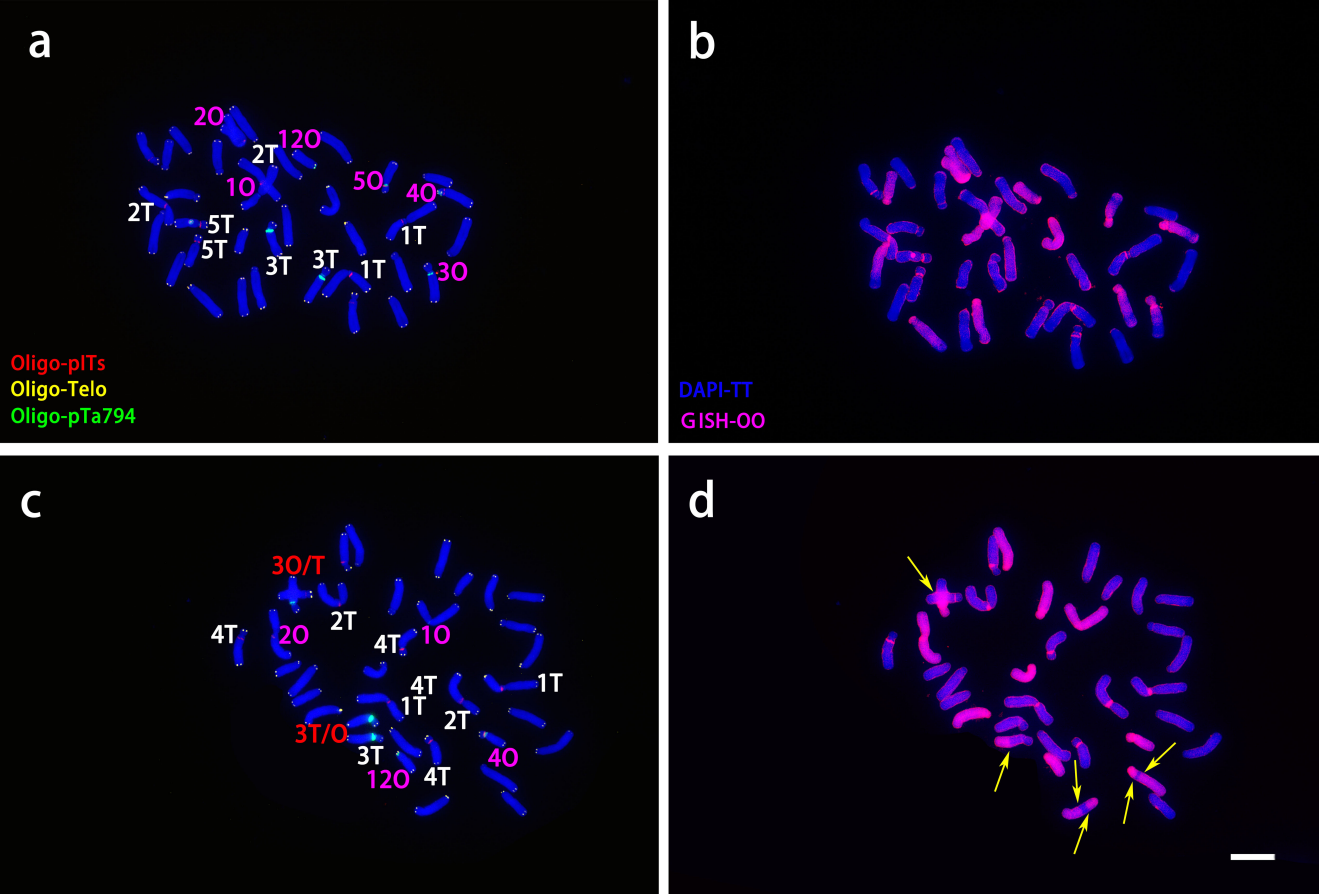
ND-FISH experiments on chromosomes of the two ‘Nymph’×*L. regale* hybrid progenies were performed using Oligo-pITS (red), Oligo-Telo (yellow) and Oligo-pTa794 (green) and as probes, showing the fluorescent signals on the chromosomes of NR-002 **(A)** and NR-019 **(C)**. GISH experiments on chromosomes of the two ‘Nymph’×*L. regale* hybrid progenies were performed with genomic DNA of *L. speciousm* ‘gloriosoides’ as a probe showing the chromosome composition of NR-002 **(B)** and NR-019 **(D)**. The yellow arrow points to the break point of the recombinant chromosomes. Chromosomes were counterstained with DAPI (blue). Scale bar 10 μm.

**Figure 8:**
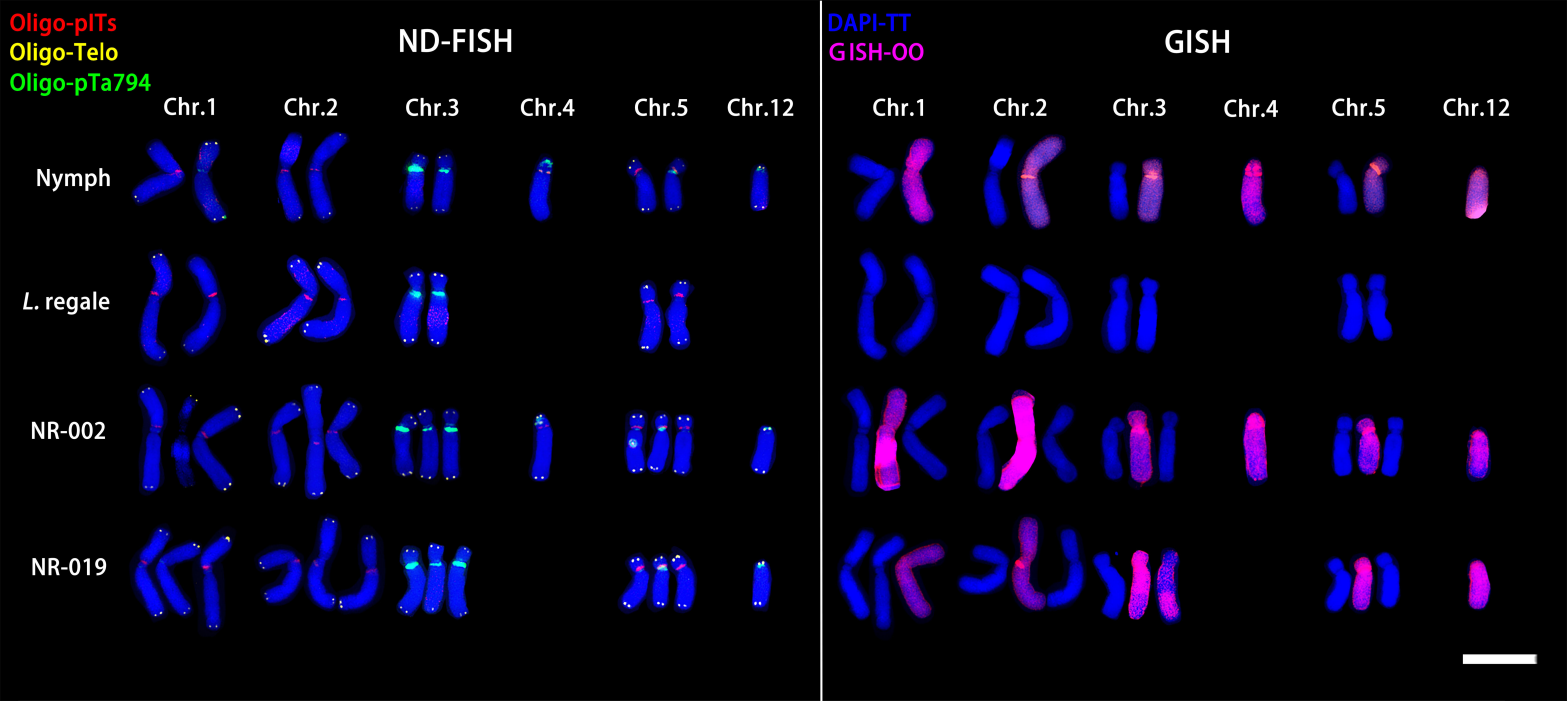
ND**-**FISH (left) and GISH (right) patterns of partial chromosomes from the maternal and paternal parents and two progenies.

ND-FISH and GISH techniques were employed to analyze the signal loci of oligonucleotide Oligo-pITS and Oligo-pTa794 in the hybrid progeny of ‘Nymph’×*L. regale* (**Figures 7**, **8**). The hybrids NR-002 and NR-019 were confirmed as true hybrids, with chromosomes from both parents clearly observed in the progeny. NR-002 was triploid, containing 36 chromosomes, while NR-019 was near-triploid, with 35 chromosomes, as cytological analysis revealed the loss of the L-genome copy of chromosome 4. Both oligonucleotide probes successfully labeled all copies of chromosomes 1, 2, 3, and 5. In both NR-002 and NR-019, these chromosomes consisted of two copies from ‘Nymph’ and one copy from *L. regale*. In NR-019, chromosome 3 showed evidence of recombination between the maternal O-genome copy and the paternal T-genome copy. Chromosome 4 in NR-002 exhibited a unique O-genome copy, which carried signals from both oligonucleotide probes, with Oligo-pTa794 signals distributed on the short arm. This particular copy originated from the maternal parent ‘Nymph’. However, this copy was absent in NR-019, likely due to loss during chromosome recombination.

## 4 Discussion

In this study, we successfully applied a combination of Non-Denaturing Fluorescence *In Situ* Hybridization (ND-FISH) and Genomic *In Situ* Hybridization (GISH) techniques to identify and characterize hybrid progeny from crosses between wild lily species and intersectional hybrids. Our research focused on three LA hybrids and three OT hybrids, as these represent the most widely studied and prevalent intersectional crosses in lily breeding. Traditionally, FISH or GISH techniques have been used independently to identify lily hybrids (Barba-Gonzalez et al., 2006; Zhou et al., 2008a; Lee et al., 2014; Zhong et al., 2022; He et al., 2022), However, these techniques are not suitable for identifying the progeny of intersectional hybrids due to the complexity of their genomic composition. While attempts have been made to combine these methods (Marasek et al., 2004; Islam et al., 2022), precise chromosome localization remains challenging due to the inability to perform both techniques simultaneously. This limitation arises from the requirement of chromosome denaturation during hybridization in traditional FISH, which can disrupt chromosomal morphology and hinder successive hybridization signals (Ren et al., 2019; Sun et al., 2022).

The success of our combined approach is primarily attributed to the use of oligonucleotide probes in the ND-FISH technique. This innovative method avoids chromosome denaturation, thus preserving morphological integrity, which is particularly valuable for successive reprobing in the same division phase (Cuadrado and Jouve, 2010). In our study, we successfully achieved the simultaneous hybridization of four distinct signals on a single slide, comprising three oligonucleotide probes and one genomic probe. This multi-signal approach enabled us to conduct a comprehensive chromosome analysis with unprecedented efficiency. Although our method employs fewer probes compared to some wheat chromosome studies that utilize up to 9 probes (Tang et al., 2016), it nonetheless yielded rich and valuable insights into lily chromosome structure and behavior.

Our findings demonstrate that oligonucleotide probes can not only effectively replace traditional rDNA probes but also offer superior sensitivity in chromosome labeling. In *L. davidii* var. *unicolor*, we detected four Oligo-pITS signals and two Oligo-pTa794 signals, and in *L. regale*, we observed eight Oligo-pITS signals and two Oligo-pTa794 signals. Our results demonstrate that the Oligo-pTa794 marking on the chromosomes of *L. davidii* var. *unicolor* and *L. regale* is consistent with previous studies using 5S rDNA probes (Wang et al., 2012; Wu et al., 2019). However, our Oligo-pITS probe revealed more signal sites compared to traditional 45S rDNA probes used in previous studies (Wang et al., 2012). Additionally, ND-FISH employs commercially synthesized oligonucleotide probes and requires shorter hybridization times, substantially reducing costs (Harun et al., 2023). Oligonucleotide probes demonstrate significant potential for lily research, especially considering the large and incompletely characterized lily genome. The prevalence of repetitive sequences in lilies, particularly satellite and tandem repeats, offers an excellent foundation for FISH probe development. Identification of short motif sequences specific to these repeats enables the synthesis of targeted oligonucleotide probes for FISH applications (Danilova et al., 2012; Fu et al., 2015; Tang et al., 2014, Tang et al., 2016). These short synthetic probes facilitate the distinction and visualization of satellite repeat subfamilies while offering several advantages over traditional probes, including consistent probe quality, cost-effectiveness, and reduced preparation time (Fu et al., 2015; Tang et al., 2016).

Building on these advantages of the ND-FISH technique, we were able to combine it with GISH, enabling precise localization of chromosomes in hybrid progeny. This combined approach allowed for early and rapid identification of hybrid chromosomes, detection of chromosomal structural variations in progeny, and inference of parental chromosome inheritance patterns (Yu et al., 2019; Wang et al., 2021, Wang et al., 2023). Firstly, the ability to precisely localize chromosomes allows for early identification of hybrid progeny. In the hybrid progeny between ‘Richmond’ and *L. davidii* var. *unicolor*, we observed that both aneuploid offsprings contained two special chromosome 3, one copy from the maternal parent and the other from the paternal parent. Similarly, in the hybrid progeny between ‘Nymph’ and *L. regale*, we found that both contained the distinct O-genome copy of chromosome 5 from ‘Nymph’. These distinct chromosomes labeled by multiple fluorescent signals can be directly utilized to authenticate hybrids and provide important tools for future research and applications (An et al., 2019; Ren et al., 2022). Secondly, our approach enables the detection of chromosomal structural variations in hybrid progeny. In RD-003, chromosome 3 from the paternal parent exhibited a different morphology compared to the paternal plant, indicating recombination events during meiosis. And analysis of NR-019 identified it as a near-triploid with 35 chromosomes, due to the loss of one O-genome copy of chromosome 4 from the maternal parent ‘Nymph’. These findings demonstrate the power of our technique in detecting structural changes and offering insights into the genetic diversity of hybrid progeny. These observations demonstrate the power of our technique in identifying structural changes and providing insights into the genetic diversity of hybrid progeny. Thirdly, and perhaps most significantly, this combined approach provided crucial insights into the mechanisms of 2n gamete production, which is essential for understanding polyploidization in lily breeding. Our hybridization results between ‘Richmond’ and *L. davidii* var. *unicolor* demonstrated near-pentaploid and near-triploid aneuploid progeny, consistent with previous findings (Lim et al., 2003; Zhou et al., 2012). The near-pentaploids likely resulted from near-triploid egg cells from ‘Richmond’ combining with diploid pollen grains from *L. davidii* var. *unicolor*. analyses showed that RD-003 contained 12 L-genome chromosomes from the maternal parent and 46 A-genome chromosomes from both parents. The 2n gametes produced by ‘Richmond’ were likely through the First Division Restitution (FDR) mechanism, while the mechanism in *L. davidii* var. *unicolor* remains unclear, though Second Division Restitution (SDR) was ruled out, because the two copies of chromosome 3 with inconsistent Oligo-Pta794 brightness both appeared in the progeny RD-003. The hybridization between ‘Nymph’ and *L. regale* was more successful, resulting in a greater number of triploid and near-triploid progeny. This was primarily attributed to the combination of 2n egg cells from the maternal ‘Nymph’ with n pollen grains from *L. regale*. NR-002 contained 12 O-genome chromosomes with centromeres (Chung et al., 2013), whereas NR-019 had only 11, missing one. The mechanism for 2n gamete production in the OT lily ‘Nymph’ was also FDR. Our findings are consistent with previous studies, further confirming that FDR is the primary mechanism for 2n gamete production in lilies (Barba-Gonzalez et al., 2004; Barba-Gonzalez et al., 2005). Therefore, our findings suggest that 2n gamete production is not limited to diploid interspecific F1 hybrids such as OA, LA, OT, etc (Barba-Gonzalez et al., 2004; Zhou et al., 2008b; Chung et al., 2013; Zhang et al., 2017). We observed that allotriploids, homoploid diploids and wild lilies can also generate a small amount of 2n gametes (Kondo et al., 2022; Xie et al., 2022). Such 2n gametes can be directly utilized for designed breeding, as in potato (Carputo et al., 2000; Carputo and Barone, 2005), Orchidaceae (Zeng et al., 2020; Kondo et al., 2022) and other species (Crespel and Gudin, 2003; Lai et al., 2015; Jiang et al., 2021).

Despite these significant advantages, it is important to note that the signals generated by our probes were not sufficient to identify all chromosomes in lily somatic cells. This limitation underscores the necessity for further research and probe development. Future efforts should focus on enhancing probe specificity and signal strength to achieve comprehensive chromosome identification in lilies, developing more specific probes to further improve the efficiency and accuracy of lily chromosome analysis, and exploring the potential of repetitive sequences in lilies, particularly satellite and tandem repeats, for FISH probe development

In conclusion, the combination of ND-FISH and GISH techniques, particularly with the use of oligonucleotide probes, provide powerful tools for lily cytogenetic research through precise chromosome localization. This approach enables early identification of hybrid chromosomes, detection of chromosomal structural variations, and inference of 2n gamete formation mechanisms. Through accurate identification and early screening of hybrid progeny, we can more effectively study the genetic mechanisms of interspecific hybridization. As we continue to refine these methods, we anticipate significant advancements in our understanding of lily genome organization and evolution, which will ultimately contribute to more efficient and targeted lily breeding strategies.

## 5 Conclusions

This present study is the first report on the application of three new oligonucleotide probes for chromosome labeling in lilies, providing new insights for cytogenetic research in lilies. Here we successfully analyzed the hybrid progeny between two wild lilies and LA and OT hybrid lilies using ND-FISH and GISH techniques. The obtained results clearly demonstrate that this hybridization strategy is feasible for achieving genetic recombination and innovation through the use of 2n gametes. By overcoming reproductive barriers and enabling genetic recombination and innovation, the combined use of ND-FISH and GISH techniques enable more efficient hybrid identification and provide powerful tools for lily breeding. Through precise identification and early screening of hybrid progeny, we can more effectively develop new lily varieties with enhanced traits and expanded genetic diversity. Future research should continue to optimize these techniques and develop more specific probes to further improve the efficiency and accuracy of lily breeding.

## Data Availability

The raw data supporting the conclusions of this article will be made available by the authors, without undue reservation.
